# Homeschooling for Quarantined Residents: A Virtual Back to the Basics Curriculum

**DOI:** 10.7759/cureus.11824

**Published:** 2020-12-01

**Authors:** Anthony Sielicki, Jennifer White, Mitchell Berman, Belinda Lao, Megan Stobart-Gallagher

**Affiliations:** 1 Emergency Medicine, Thomas Jefferson University Hospital, Philadelphia, USA

**Keywords:** online medical education, post grad medical education, virtual learning environment, quarantine

## Abstract

Introduction

The COVID 19 pandemic resulted in local and institutional restrictions with significant effects on the clinical environment for graduate medical education, displacing residents from non-emergency medicine (EM) based rotations. Additionally, resident physicians considered patients under investigation (PUI) were furloughed from clinical practice. The necessity for supplemental learning in a virtual setting prompted the development of an online homeschooling curriculum that incorporated back to the basics textbook learning, application, and retention via virtual sessions for the quarantined and furloughed learners.

Methods

An online homeschooling curriculum was developed to replace the cancelled clinical experiences for EM residents and for those who were quarantined utilizing Google Classroom and Zoom teleconference software. After completion of their quarantine or return to normal rotation schedule, residents were asked to evaluate the homeschooling curriculum using an anonymous survey.

Results

A total of 12 residents participated in the homeschooling program over eight weeks during the spring of 2020. Of the nine residents surveyed, 88.8% percent felt the homeschooling added to their knowledge of EM, 100% found the online format easy to use, and 88.8% stated it helped maintain a sense of social connection to peers and faculty.

Conclusion

An online homeschooling program was considered an effective means of providing an opportunity for synchronous and continuous education for EM resident physicians. This program could be sustainable long term to fill in knowledge gaps or supplement remediation in emergency resident education, post pandemic.

## Introduction

The novel coronavirus disease-19 (COVID-19) pandemic has vastly changed the ways in which core content and knowledge is delivered to emergency medicine (EM) residents. Typical educational methods, such as live resident didactics, morning reports, and journal clubs, are inherently social in nature and call for large group gatherings to be executed. Many institutions have banned such gatherings in honor of social distancing. Exposure to or contracture of COVID-19 in the clinical environment has forced many residents and faculty to quarantine for extended periods of time, posing further barriers to typical educational methods. The pandemic has also led to major alterations or to the cancellation of several non-EM clinical rotations that are core to the foundation of EM education, including emergency medical services (EMS), anesthesia, ultrasound, and elective rotations. 

Online methods of education seem ideally poised to fill in these gaps. Virtual learning has been used extensively in undergraduate medical education, ranging from recorded lectures to online patient experiences [1]. Even during the COVID-19 pandemic, undergraduate medical educators have been quick to adapt to new forms of education, with methods stretching from tele-teaching rounds [2,3], virtual simulation sessions with standardized patients [4], and have suggested using augmented reality to help deliver procedural education [5]. However, the use of online education within graduate medical education has not been as well described. Within EM resident training, virtual learning has been used as a method of onboarding interns [6] and as a means of providing asynchronous learning and assessment [7]. During the COVID-19 pandemic, educational experts have urged for adaptation and migration of typical education techniques like weekly didactics to online formats [8], but few solutions have been raised to augment the training experience for quarantined residents. 

The virtual back to basics homeschooling curriculum was developed to provide a structured learning environment for quarantined residents during a time when clinical and experiential learning was not possible. We define quarantined residents as all of those residents deemed patients under investigation (PUI) and those displaced from non-EM clinical rotations. The primary goal of the homeschooling curriculum was to provide a small group setting to fill in gaps in knowledge regarding the core content of EM. Secondary outcomes included creating a sense of social connection to peers and faculty in a time of potential isolation.

## Materials and methods

Homeschooling was initiated over a period of eight weeks in the spring of 2020 for quarantined residents at our EM residency program in Philadelphia, Pennsylvania - an epicenter for the pandemic. Attendance was required for residents who had rotations cancelled and if feeling well enough, for those residents on home quarantine due to suspected or confirmed infection with COVID-19. All other residents in the EM residency were encouraged to attend. Our faculty and advanced practice providers (APP) were also invited to attend as part of their continuing education in the practice of EM. Sessions were typically attended by five to seven residents, two to three faculty members, one to two APPs and lasted approximately two hours in length. Residents who were required to attend homeschooling spent an average of two to four weeks in the program. Upon finishing their quarantine or when schedules allowed them to return to a rotation unaffected by COVID-19, they were no longer required to attend homeschooling. 

The homeschooling curriculum was organized to provide structured daily educational activities to quarantined residents. The ultrasound division held a weekly tape review and discussion of emergency ultrasound literature on Mondays. Previously scheduled weekly didactics continued to be held routinely on Wednesdays. To comply with social distancing requirements, both of these activities were migrated to synchronous, virtual formats using Zoom conferencing software. The remaining three days per week were structured to focus on core content to grow foundational knowledge and application. Table 1 shows a typical weekly schedule. 

**Table 1:** Typical weekly schedule

Day of the Week	Educational Activity
Monday	Ultrasound Conference and Tape Review
Tuesday	Homeschooling: Core Content and Advanced Discussion
Wednesday	Resident Conference
Thursday	Homeschooling: Core Content and Advanced Discussion
Friday	Homeschooling: Mock Oral Boards and/or Virtual Simulation

Educational content was preassigned and uploaded to a Google Classroom folder that was shared with all participants and monitored by the chief residents and Graduate Medical Education (GME) leadership. This content included chapters from the Rosen’s Emergency Medicine textbook, Roberts and Hedges textbook, research articles, and links to Free Online Access Medical Education (FOAMEd) resources. Tele-video conferencing via Zoom was used to bring residents and faculty together to discuss the reading and to complete an educational activity. Homeschooling sessions utilized a variety of education techniques, including asynchronous learning, flipped-classroom, small-group discussion, mock oral boards, virtual simulation, and story-telling. Table 2 describes a sample of the readings and educational activities. 

**Table 2:** Example of homeschooling educational activities

Session	Reading	Activity
1	Rosen Chapter 2: Mechanical Ventilation	Small group discussion and storytelling: Describe the condition of the last patient you placed on a ventilator, which ventilation strategy you used and why. What is the mode of ventilation you would choose for a patient that is fully sedated and paralyzed? Why? Which mode should you never choose? Have you ever intubated an asthmatic? What specific considerations are there in ventilator strategies for an asthmatic? Describe one way you have been asked to troubleshoot a ventilator problem and your algorithm for doing this.
2	Rosen Chapter 80: Implantable Cardiac Devices	Case presentation: A patient who is pacer dependent secondary to AV node ablation for atrial fibrillation was using a snow blower and passed out.
3	Rosen Chapter 6: Shock Seif D, Perera P, Mailhot T, et al. Bedside ultrasound in resuscitation and the rapid ultrasound in shock protocol. Crit Care Res Pract. 2012;2012:503254. doi: 10.1155/2012/503254. Epub 2012 Oct 24.	Small group discussion and storytelling: Have you ever treated a patient with shock? What type of shock was it and how did you know? What is a situation where it might be different to tell one type of shock from another? What can you do to distinguish them? Has there been a case where you weren’t sure which type you were dealing with or might have been dealing with more than one type of shock? What did you do?
4	Permpikul C, Tongyoo S, Viarasilpa T, et al. Early Use of Norepinephrine in Septic Shock Resuscitation (CENSER). A Randomized Trial. Am J Respir Crit Care Med. 2019 May 1;199(9):1097-1105. doi: 10.1164/rccm.201806-1034OC. Swaminathan A. REBELEM. Occult Causes of Non-Response to Vasopressors https://rebelem.com/occult-causes-of-non-response-to-vasopressors/. July 13, 2017. Accessed April 4, 2020.	Case presentation: 82 year old female presents from a nursing home with altered mental status. Paperwork from the facility shows a past medical history of congestive heart failure, coronary artery disease, chronic kidney disease, gastrointestinal bleeding, and hospitalization 1 month ago for urosepsis. Small group discussions focusing on: EBM management of septic shock and early vasopressor use
5	Murthy S, Gomersall CD, Fowler RA. Care for Critically Ill Patients With COVID-19. JAMA. Published online March 11, 2020. doi:10.1001/jama.2020.3633 Poston JT, Patel BK, Davis AM. Management of Critically Ill Adults With COVID-19. JAMA. Published online March 26, 2020. doi:10.1001/jama.2020.4914	Virtual simulation - Using a sample case from the Full Code application (https://full-code.com/), a virtual simulation of a patient with fever and cough presents to the emergency department from the airport after travel to an area endemic with COVID-19. Focus on staff safety with PPE discussion, resuscitation of critical COVID-19 PUI.
6	No assigned reading	Mock oral boards - Faculty facilitators planned mock oral boards cases, with the seniors running them. The remainder of the group were given critical actions and the case in order to provide feedback on their performance and evaluation of whether critical actions were executed. Cases used included a tricyclic antidepressant overdose, as well as pericarditis.

Faculty and senior residents were responsible for pre-assigning the readings, planning educational activities, and serving as facilitators for the discussions and activities. Senior residents were included as teachers in this format to supplement their prior experience as clinical educators, which was also now lacking due to cancelled medical student rotations. Using residents as educators in this setting was guided by the Accreditation Council for Graduate Medical Education (ACGME) core competency that states residencies should include the role of the physician as an educator to "educate patients and families" and to "facilitate the learning of students and other health care professionals [9]. They collaborated with faculty to create post-reading quizzes and ran case-based scenarios during the scheduled sessions.

Twelve residents (three postgraduate year (PGY)-3, three PGY-2, and six PGY-1) participated in virtual homeschool over the eight weeks. They completed an evaluation utilizing a Google-from based survey. The survey consisted of one demographic question, seven questions inquiring about the ease, content, satisfaction, and social impact of the homeschooling curriculum, and two open-ended questions for general feedback (Table 3).

**Table 3:** Assessment survey of the homeschooling curriculum 1- Strongly disagree, 2- Disagree, 3- Neither agree nor disagree, 4- Agree, 5- Strongly agree

Assessment Question	Mean (STD)
The google classroom and google form-based homeschooling curriculum was easy to use.	4.78 (0.44)
The homeschooling curriculum made me feel engaged in learning.	4.22 (0.97)
The homeschooling curriculum added to my knowledge of emergency medicine.	4.56 (0.72)
The content of the homeschooling curriculum was enjoyable.	4.11 (1.05)
The online format of the homeschooling curriculum provided a means of social connection with my peers and faculty.	4.56 (1.05)
The online format of the homeschooling curriculum should be continued, even after the pandemic ends.	3.33 (1.58)
(Optional for those who crafted homeschooling lessons) Preparing homeschooling lessons made me feel more confident as a medical educator.	4.0 (0.71)
If you did not have the homeschooling curriculum, how would you have spent your time? How would you have kept to a studying schedule?	
Please provide any other thoughts or feedback regarding the homeschooling curriculum.	

## Results

The survey was emailed to the twelve residents who engaged in the initial eight weeks of the homeschooling curriculum and was completed by nine, yielding a response rate of 75%. Of the respondents, five of nine were PGY-1, three were PGY-2, and one was a PGY-3. All of the respondents agreed or strongly agreed that the online Google Classroom and Zoom-based format was easy to use. Eight of nine respondents believed that it added to their knowledge base of EM. Respectively, 88.8% and 78% felt engaged during homeschooling sessions and enjoyed the content of the curriculum. Eight of nine residents felt homeschooling assisted in maintaining a social connection to their peers and faculty. For the senior residents (n=4) who planned educational content and led small group discussions, 80% agreed or strongly agreed that it added to their confidence as a medical educator. Only 55.5% of respondents wanted the online homeschooling curriculum to continue after the pandemic ends. 

General feedback from the free text questions on the survey reflected that residents enjoyed the daily structure provided by the homeschool curriculum. Many wrote that the small-group format provided accountability and allowed them to be more productive and learn more than if no structure had been provided for them (i.e. left to plan their own studying during this time period). They appreciated the brevity of the homeschooling sessions and the focus on foundational knowledge of EM. Critiques of the program were that reading materials should be provided earlier to provide more time for reading, and that some content should be targeted towards high-level learners in order to make it more engaging for senior residents. 

## Discussion

These results demonstrate that homeschooling was generally well received by EM residents. Based on survey data, the primary goal of providing a structured learning environment was successful, as a majority responded it added to their knowledge base of EM and held them accountable for learning. The secondary goals of keeping residents engaged and building resident confidence as medical educators were also accomplished. Providing a means of social connection to faculty and peers was attained, and is important as studies of prior incidents of epidemics and quarantine have demonstrated rates of depression to be >30% in affected populations [10]. Contrary to these largely positive results, only a slight majority responded that the homeschooling program should be continued after the pandemic ends. This may be due to fear of over-burdening after returning to clinical rotations, or perhaps due to a preference for in-person learning.

This study builds on the knowledge that online education, including methods using Google-based software, can be used to augment EM resident education [7]. It not only complies with educational expert opinion that virtual learning needs to occur during the COVID-19 pandemic [8], but also offers a solution on how to supplement educational experiences for residents in quarantine or affected by canceled clinical rotations. 

The sample size for this study is relatively small, with only 12 residents of our 50 resident program who participated in homeschooling activities thus far. It may have been difficult to anonymize due to the small sample size, which may have prevented residents from completing the evaluation. While this may not give a substantial amount of data, it did make for smaller, more focused, and interactive homeschooling sessions. Of note, half of the cohort of the homeschooling population (six of twelve residents) were PGY-1. Likewise, our survey results were predominantly completed by PGY-1s (five of nine responses). This may be due to the PGY-1 schedule, which has more clinical rotations that were subjective to cancelation due to COVID-19, such as EMS and anesthesia. 

The homeschooling curriculum benefited from faculty involvement in preparation and participation in the sessions but this may be difficult for residency programs without a robust faculty group or for those emergency departments who are clinically stressed by staffing concerns. In addition, the data collected from homeschooling participants focused only on participant perceptions of medical knowledge acquisition. Future studies should be performed to assess if similar programs result in objective improvement, such as with in-training exam (ITE) scores or patient outcomes. 

Though this homeschooling curriculum was designed to fill an educational void for quarantined residents, it also has potential future uses outside of this domain. A similarly designed program may be beneficial for trainees who require academic remediation or those who struggle with applying foundational knowledge to the clinical setting. It may also be valuable in EM resident rotations that do not have dedicated didactics, such as EMS or anesthesia. Further research and a trial in these areas are also warranted.

## Conclusions

The homeschooling curriculum is an easily implementable online-learning environment that can aid in the training of residents who have been isolated by an international pandemic. It continues to be used and refined at our program, and is under consideration as an opportunity to expand synchronous virtual learning experiences for applications in the future such as off-service rotations without planned didactics and those residents on academic remediation.
